# Bone morphogenetic protein (BMP)-9, a systemic homeostasis factor with diverse functions in the liver and beyond

**DOI:** 10.1042/BSR20253294

**Published:** 2025-12-11

**Authors:** Deeksha Rana-Seyfert, Anna Grab, Katja Breitkopf-Heinlein

**Affiliations:** 1 Department of Surgery, Medical Faculty Mannheim, Heidelberg University, 68167 Mannheim, Germany.

**Keywords:** endoglin, TGF-beta, ALK1, BMP-9, homoeostasis, liver

## Abstract

Bone Morphogenetic Protein-9 (BMP-9), a circulating cytokine belonging to the TGF-β superfamily, is increasingly recognized as a critical regulator of tissue homeostasis with diverse roles in both health and disease. This review recapitulates current knowledge regarding BMP-9, shifting focus beyond its traditional association with bone formation to highlight its significant influence on physiology and pathology in the liver but also in other tissues and organs. We discuss how BMP-9 exerts intricate control over various cell types, affecting key processes such as angiogenesis, macrophage polarization, and regeneration. Intriguingly, while in the liver, BMP-9 contributes to the maintenance of sinusoidal endothelial cell quiescence and Kupffer cell identity, dysregulation of its signaling can as well promote fibrosis. The complexity of BMP-9 function is further compounded by its cross-talks with other signaling molecules like TGF-β, as well as non-Smad pathways. In this review, we summarize current knowledge about the functions of BMP-9 in humans and discuss its therapeutic potential.

## Introduction

Bone morphogenetic protein (BMP)-9, also termed growth and differentiation factor (GDF)-2, is a secreted cytokine, belonging to the TGF-β super-family. It is constitutively synthesized in healthy individuals and circulates with the blood stream in an active conformation. Within the BMP subfamily, BMP-9 and -10 are forming their own subgroup, meaning that BMP-9 and -10 are not closely related to any other BMP or to any other member of this family of cytokines. BMP-9 was long considered liver-specific while BMP-10 seemed to be only relevant in the heart. This distinction can no longer be maintained at present, as BMP-10 for example is apparently also expressed in the liver, also circulates in the blood, and can even form functional heterodimers with BMP-9 [1]. However, there is much more information available regarding BMP-9 than -10 and this review will therefore focus mainly on BMP-9.

The TGF-β type I receptor ALK1 (ACVRL1) binds exclusively to these two BMPs, while other type I receptors have overlapping affinities [2]. BMP-9’s ability to signal via canonical Smad pathways or noncanonical pathways (e.g., MAPK) depends on receptor availability, such as the type I receptors ALK1 or ALK2, which may influence tissue homeostasis as well as target genes critical for disease progression. This review summarizes the current knowledge about BMP-9’s diverse functions in health and disease, emphasizing BMP-9 as a potential therapeutic target in humans in the future.

## Sources of BMP-9 in the body

Already in 2012, Bidart et al. postulated that BMP-9 is produced by hepatocytes [3]. While the liver has since then clearly been established to be the main organ of origin of BMP-9 [4,5], the cellular source is nowadays not any more considered to be the hepatocyte. In 2017 we demonstrated that hepatic stellate cells (HSCs) are the main producers of BMP-9 in both mouse and human liver, with hepatocytes showing no detectable expression [4], and this was further supported in human liver published by us in 2023 [6]. In 2000 Miller et al. reported again that BMP-9 is produced in the liver by nonparenchymal cells, but not hepatocytes [5]. The observation of HSCs being the primary source of BMP-9 was recently confirmed again by Dianyuan et al. [7]. Liu et al. published in 2023 that BMP-9 can be expressed by hepatocytes and macrophages of the liver in mice [8]. However, on the one side, these expression levels were not directly compared with HSCs, and on the other hand, they may represent species differences between mice and humans. Basically, BMP-9 is produced and secreted as a pro-protein version, which is already fully active [3,9]. Research has demonstrated that BMP-9 mRNA is expressed by both quiescent and activated HSCs [4], suggesting that HSC-derived BMP-9 might be relevant under diverse physiological as well as pathological conditions like liver fibrosis.

Recent investigations have raised the possibility that significant *in vivo* expression of BMP-9 may not be limited to HSCs (or the liver in general), but could also occur in other nonhepatic cell types. Indeed, Morine et al. reported that cardiac fibroblasts produce BMP-9 and that this is highly relevant *in vivo* by improving cardiac function in heart failure [10]. Our previous work has shown that some unknown cell type in the small intestine can also express BMP-9 in humans, that this expression decreased in diabetic patients and that it is positively correlated with intestinal E-Cadherin expression [6], implying a role for BMP-9 in stabilizing the gut barrier. Luo and colleagues reported BMP-9 expression (RNA as well as protein) in muscle and adipose tissue, and both were reduced in patients with newly diagnosed type 2 diabetes [11]. Furthermore, He et al. revealed high BMP-9 expression in the hypothalamus, which was significantly reduced in obese mice [12]. In line with these findings, Ogawa et al. demonstrated that BMP-9 is expressed in rat brain [13]. We further described that IL-6 was able to directly up-regulate BMP-9 expression in human liver-derived mesenchymal stroma cells *in vitro* [14]. In conclusion to these findings, it might be speculated that under certain conditions, several different cell types or tissues in the body might be able to up-regulate their local BMP-9 expression and might thereby contribute to diverse physiological or pathological processes *in vivo*. However, this and the question of which source of BMP-9 might finally contribute most to the circulating, systemic levels of BMP-9 in humans still need to be further investigated. In any case, local as well as systemic levels of BMP-9 exist, which means that BMP-9 is most likely active throughout the body and therefore likely to have cross-organ effects in health and disease.

## BMP-9 serum levels in health and disease

### Control of BMP-9 expression

Human serum levels of BMP-9 in healthy individuals measured by ELISA are reported to range around 50–70 pg/ml; however, the numbers vary extensively between the diverse measurement techniques (see Table 1).

**Table 1:** Summary of published changes in BMP-9 serum- or plasma levels in humans under diverse conditions in health and disease

Literature	Type of patients		n-numbers (e.g., healthy vs diseased)	Change of BMP-9 levels	Method used
Yang et al., 2024 [15]	**Age: 20–40**	**Age: 60–80**			
	67.40 ± 6.58	65.16 ± 6.38	75 vs 32	Down with age	ELISA (Hengyuan Biotechnology Co., Ltd, Shanghai, China)
Li et al., 2018 [16]	**Age: ca. 25–95 years**				
	Ranging from ca. 5 up to ca. 350		60 (34 healthy + 26 fibrosis)	No correlation with age	ELISA (self-designed)
Yang et al., 2024 [15]	**Female**	**Male**			
	68.10 ± 5.90	64.84 ± 6.57	63 vs 103	Higher in females	ELISA (Hengyuan Biotechnology Co., ltd, Shanghai, China)
[Xu et al., 2017] [17]	**Female**	**Male**			
	54.75	51.38	205 vs 157	No significant gender difference	ELISA (R&D systems)
Yang et al., 2024 [15]	**Underweight**	**Obese**			
	73.00 ± 4.65	65.29 ± 5.23	5 vs 10	Down with obesity	ELISA (Hengyuan Biotechnology Co., Ltd, Shanghai, China)
[Xu et al., 2017] [17]	**Noncentral obesity**	**Central obesity**			
	61.58	50.93	125 vs 237	Down with central obesity	ELISA (R&D systems)
Drexler et al., 2023 [6]	**Adipose, no diabetes**	**Adipose with diabetes**			
	77.47 ± 25.74	74.18 ± 25.01	7 vs 13	No significant change with (additional) diabetes	ELISA (SimpleStep®; Abcam)
[Xu et al., 2017] [17]	**Normal lipids**	**Dyslipidemia**			
	75.72	49.73	92 vs 270	Down with dyslipidemia	ELISA (R&D Systems)
[Xu et al., 2017] [17]	**Normal BP**	**Hypertension**			
	59.75	49.72	169 vs 193	Down with hypertension	ELISA (R&D Systems)
Huang et al., 2018 [18]	**Normal BP**	**Hypertension**			
	77.21	46.20	93 vs 132	Down with hypertension	ELISA (R&D Systems)
Liu et al., 2019 [19]	**Healthy**	**Hypertension**			
	123.3 (45.5–178.6)	53.2 (31.8–62.8)	121 vs 131	Down with hypertension	ELISA (R&D Systems)
[Xu et al., 2017] [17]	**Non-IR**	**IR**			
	68.48	49.17	174 vs 143	Down with insulin resistance (IR)	ELISA (R&D Systems)
[Xu et al., 2017] [17]	**Healthy**	**Metab. syndrome**			
	78.39	44.31	147 vs 215	Down with metabolic syndrome	ELISA (R&D Systems)
Luo et al., 2017 [11]	**Healthy**	**T2DM**			
	73.1	40.4	121 vs 159	Down with diabetes	ELISA (R&D Systems)
Hao et al., 2022 [20]	**Healthy**	**T2DM**			
	167.50 ± 58.49	95.01 ± 34.03	30 vs 30	Down with diabetes	ELISA (Cloud-Clone Corp., Wuhan, China)
Hao et al., 2022 [20]	**Healthy**	**NAFLD**			
	167.50 ± 58.49	90.50 ± 28.50	30 vs 30	Down with NAFLD	ELISA (Cloud-Clone Corp., Wuhan, China)
Yang et al., 2024 [15]	**Healthy**	**NASH at risk**			
	70.32 ± 3.70	58.13 ± 2.82	66 vs 32	Down with NASH	ELISA (Hengyuan Biotechnology Co., ltd, Shanghai, China)
Li et al., 2018 [16]	**Healthy**	**Liver fibrosis**			
	ca. 20 ( < 50)	ca. 200	56 vs 52	Up with liver fibrosis	ELISA (self-designed)
Owen et al., 2020 [21]	**Healthy**	**Liver cirrhosis**			
	ca. 50	ca. 300	21 vs 71	Down with decompensated cirrhosis	ELISA (self-designed)
Olsen et al., 2014 [22]	**Healthy**	**Multiple myeloma**			
	110 (median)	176 (median)	58 vs 138	Up in multiple myeloma patients	Multiplex assay (magnetic beads, Millipore)
Li et al., 2021 [23]	**Control**	**Venous malformations**			
	72.55 ± 73.17	13.10 ± 25.36	70 vs 66	Down with venous malformations	ELISA (RayBiotech, ELH-BMP9)
David et al., 2008 [24]	**No HHT patients**	**HHT patients**			
	6200 ± 600	5000 ± 700	20 vs 20	No significant change with HHT	BRE luciferase reporter assay in relation to rec. BMP-9 protein
Dunmore et al., 2023 [25]	**Healthy**	**Covid‐19 infection**			
	ca. 95	ca. 50	29 vs 49	Down with corona infection	ELISA (self-designed)
Liu et al., 2019 [19]	**Healthy**	**Coronary heart disease**			
	123.3 (45.5–178.6)	55.0 (24.9–71.4)	121 vs 78	Down with coronary heart disease	ELISA (R&D systems)
Morine et al., 2018 [10]	**Healthy**	**Heart failure (HF**)			
	ca. 3 times increased in HF*		10 vs 45	Up with heart failure	ELISA (R&D Systems)
Bai et al., 2024 [26]	**Healthy**	**Sepsis**			
	89,46 ± 63,95	23,51 ± 12,28	30 vs 68	Down with sepsis	ELISA (R&D Systems)
Bai et al., 2024 [26]	**Sepsis patients without shock**	**Sepsis patients with shock**			
	26,95 ± 19,63	13,66 ± 9,26	32 vs 36	Down with septic shock	ELISA (R&D Systems)
Bai et al., 2024 [26]	**Sepsis patients survivors**	**Sepsis patients nonsurvivors**			
	27,26 ± 15,10	12,54 ± 6,02	47 vs 21	Down in nonsurvivors of sepsis	ELISA (R&D Systems)
Li et al., 2021 [23]	**Healthy**	**Sepsis**			
	ca. 190	ca. 60	10 vs 10	Down with sepsis	ELISA (R&D Systems)

* original numbers were not given.

BP, blood pressure. IR, insulin resistance. T2DM, type 2 diabetes mellitus. NAFLD, non-alcoholic fatty liver disease. NASH, non-alcoholic steatohepatitis. HHT, hereditary hemorrhagic telangiectasia.

The main function of BMP-9 *in vivo* seems to be to stabilize the differentiated, functional state of cells and tissues and thereby keep homeostasis up. However, surprisingly little information is available about possible regulators of this constitutive expression. Um et al. described that cold exposure of mice can induce hepatic BMP-9 expression by activating CREB (cAMP response element binding protein) and CBP (CREB-binding protein) [27]. Either overexpression of CREB or norepinephrine treatment increased BMP-9 promoter activity. These results indicate that CREB induces BMP-9 promoter activity by acting on CRE (c-AMP response element). A cAMP-regulated expression of BMP-9 was recently reproduced by Bendixen et al. in HSC [28].

Our *in vitro* study showed that IL-6 can directly up-regulate BMP-9 expression in hepatic myofibroblastic cells [4]. It was further described that *in vivo*, in mice, hepatic BMP-9 expression is up-regulated in a model of nonalcoholic steatohepatitis (NASH induced by MCD diet), and this is accompanied by enhanced hepatic IL-6 expression [29]. Nevertheless, it remains to be investigated if IL-6 is indeed an important inducer of BMP-9 also *in vivo* in humans. As stated above, down-regulation of either hepatic BMP-9 expression or reduction of the circulating serum levels of BMP-9 protein seems to generally occur during conditions of wound healing and regeneration [4] as well as with increasing age, at least in humans [15], or in pathologic conditions like adiposity or diabetes (see below). But how is this down-regulation mediated, and what are the factors that counteract the constitutive expression? In our previous work, we found that LPS, although it induces IL-6, e.g., in liver sinusoidal endothelial cells (LSECs) [14], can directly and significantly down-regulate BMP-9 expression in cultured mouse HSC *in vitro* [4]. The actions of LPS and IL-6, at least in the liver, and their relation to BMP-9 signaling are highly cell-type specific. While in LSECs, BMP-9 antagonized LPS-mediated responses, it enhanced pro-inflammatory responses in macrophages; e.g., it enhanced LPS-mediated induction of IL-6 in macrophages but reduced LPS-mediated IL-6 expression in LSECs [14]. It remains to be analyzed how these complex interactions act on LPS effects *in vivo* and if there is indeed a significant negative correlation between serum LPS and BMP-9 levels *in vivo* in humans.

Bendixen et al. reported that in a mouse model of MASH, BMP-9 expression in HSCs gets down-regulated through reduced cAMP-producing signaling [28]. Already in 2015, it was reported that the GDF2 promoter can be silenced by methylation in ovarian cancer patients, resulting possibly in tumor promotion [30].

In summary, the BMP-9 regulatory factor(s) *in vivo* need to be analyzed in more detail in order to understand how BMP-9 levels are controlled under physiological and pathological conditions.

### Changes of BMP-9 levels in disease conditions

See Table 1 for a summary of changes of BMP-9 levels that were reported in human serum or plasma in health or disease.

Interestingly, circulating BMP-9 levels might be higher in women than in men and may decrease with age in humans [15]. In contrast, in mice, hepatic BMP-9 expression was in one study reported to increase with age [8]; however, serum levels were not analyzed in this study. Another study investigated a cohort of human liver fibrosis patients and did not find any correlation of BMP-9 serum levels with the age of the patients [16]. Higher levels of serum BMP-9 in female patients were also reported by Xu et al. [17], but in this cohort of patients, this difference did not reach significance. Therefore, a general age- and/or gender-dependent difference of BMP-9 serum levels in healthy human subjects remains to be supported further.

**Figure 1:**
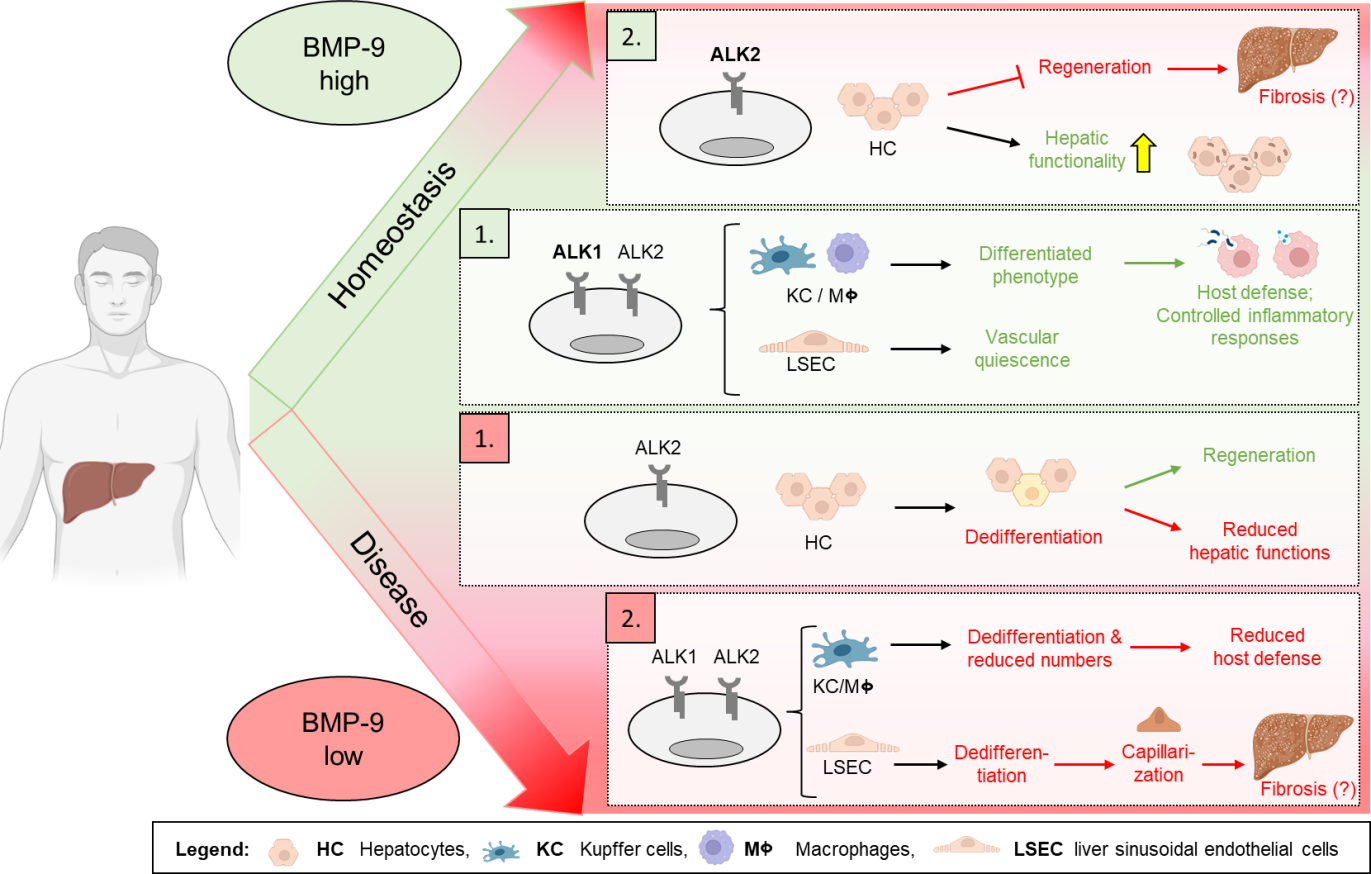
Schematic summary of the proposed functions of BMP-9 in the liver demonstrating that high as well as low levels can both lead to fibrosis, but through different cellular mechanisms. The central part of the image (colored green) shows the optimal state of BMP-9 equilibrium: BMP-9 first binds to the ALK1 receptor (due to highest affinity) and at higher levels also to ALK2 (with lower affinity), maintaining healthy, differentiated states of LSECs, KCs, and macrophages. In this healthy state, hepatocytes exhibit normal hepatic functionality but reduced regeneration capacity. The upper part of the figure depicts the scenario where too much BMP-9 finally prevents that hepatocytes can switch from their functional to the regenerative program. In this setting the regenerative, wound-healing responses of the parenchyme would be reduced and any liver-damaging factor may cause increased liver fibrosis. Conversely, the bottom part of the figure shows the effects of low BMP-9 levels: if the levels drop, this will first affect signaling via the ALK2 receptor, allowing regeneration to occur, but with the cost of reduced hepatic function. As BMP-9 levels decrease further, signaling via ALK1 will also get reduced leading to decreased KC dedifferentiation and possibly compromised host defense. Simultaneously, LSECs dedifferentiate, leading to capillarization of the sinusoids which finally results again in enhanced liver fibrosis. Implying that both, too high as well as too low levels of BMP-9 may equally promote liver fibrogenesis, although via different cellular effects. The figure was created using Biorender®.

Overall, in humans, the BMP-9 concentration in serum decreases with pathologic conditions like obesity, hypertension, insulin resistance, fatty liver disease, type II diabetes as well as coronary heart disease, venous malformations, COVID-19 infection, and sepsis [11,15,17 20,23,25,26]. In the vascular disease hereditary hemorrhagic telangiectasia (HHT), levels decreased as well, but this did not reach significance level in one study [24]. In conclusion, there seems to exist an optimal, intermediate level of circulating BMP-9 which is generally associated with good health conditions, especially regarding hepatic functionality and host defense abilities. Strongly increased levels might, in turn, support diseases like liver fibrosis by antagonizing regenerative functions (Figure 1). In line with this assumption, BMP-9 serum levels were described to significantly increase with progression of liver fibrosis [16] and in patients with heart failure [10] or myocardial infarction [31]. If liver fibrosis progresses to cirrhosis and especially decompensated cirrhosis, BMP-9 serum levels were reported to decrease again [21]. Notably, in one study, Olsen et al. reported that the BMP-9 levels were also increased in serum of multiple myeloma patients and *in vitro* BMP-9 induces apoptosis in these cells [22].

The most important question in this context is the classical ‘hen and egg’ question: do BMP-9 levels drop as cause or consequence of an event or condition? This question and whether patients would indeed profit from therapeutic ‘correction’ of BMP-9 levels remains to be addressed further.

## Signaling of BMP-9

Like other members of the TGF-β superfamily, BMP-9 can signal via the so-called canonical pathway by activating receptor complexes that are composed of two type I and two type II receptors leading to the propagation of the signal via the BMP-Smad proteins, Smads 1, 5, and 8 [2]. In this context, the presence or absence of the high-affinity receptor ALK1 mainly dictates if BMP-9 leads to signal transduction and regulation of target genes whose promoters contain Smad binding elements (SBE). If ALK1 is absent, BMP-9 may still signal via lower affinity type I receptors like ALK2. Interestingly, BMP-9 can bind with high affinity to ALK1 alone, whereas binding to ALK2 is much more efficient in the presence of type II receptors [32]. Using surface plasmon resonance, it has further been demonstrated that BMP-9 principally binds to three different type II receptors: ActRIIB with highest affinity, followed by BMPRII and with lowest affinity ActRIIA [33]. In any case, BMP-9 can also transmit signals via noncanonical signaling, including signal transduction via MAP-kinases [34].

### ALK1 pathway

The high-affinity BMP-9 type I receptor, ALK1 (ACVRL1), a transmembrane receptor with serine/threonine kinase activity, is generally highly expressed on endothelial cells (ECs) throughout the body but is also present on other cell types like macrophages (see below), fibroblasts, or adipocytes. Mutations in ACVRL1 (or endoglin) can cause the vascular disease hereditary hemorrhagic telangiectasia type 2 (HHT2) [35], which is associated with abnormal blood vessel structures and increased risk of bleedings.

In the liver, sinusoidal endothelial cells (LSECs) as well as KCs (but not hepatocytes) are the major cell types expressing ALK1 [4,5].

#### ALK1, expressed on ECs

The presence of ALK1 significantly influences BMP-9 signaling in ECs by serving as the primary receptor for BMP-9 (and -10). ALK1 is highly expressed on ECs and exhibits specific binding affinity towards BMP-9 which leads to the initiation of the Smad1/5/8 signaling cascade, followed by the regulation of gene transcription of targeted genes like ID1 [9,36,37]. This signaling pathway promotes endothelial cell quiescence and inhibits proliferation and migration and counteracts angiogenic signals induced by bFGF and VEGF [32]. The interaction of BMP-9 with ALK1 is further enhanced by the co-receptor endoglin, which typically potentiates the endothelial BMP-9 response [37], see chapter below for details.

*In vitro* in human LSECs, BMP-9 alone did not significantly down-regulate the differentiation marker LYVE-1 [14], supporting the concept that BMP-9 counteracts the capillarization of LSECs. In line with this, LSEC-specific deletion of ALK1 in mice led to induction of proangiogenic/tip cell gene sets and arterialization of hepatic vessels [38].

Interestingly, ALK1 can also bind LDL in arterial ECs and mediate its transcytosis. This is an important feature of plaque formation during atherosclerosis development, and it seems to be completely independent of BMP-9 binding and signaling [39]. Blocking this LDL binding to ALK1 using a specific antibody that does not interfere with ligand binding but still inhibited atherosclerosis initiation and progression in mice. This points to additional ALK1 effects (at least in ECs) beyond BMP-9 (or -10) signaling.

#### ALK1 expressed on macrophages

ALK1-mediated BMP-9 signaling is essential for macrophage activity, especially in the liver’s Kupffer cells (KCs), the resident macrophages of the liver. Through a Smad-dependent process, BMP-9 is essential for KC survival and identity maintenance. When ALK1 is lost, KC differentiation is abrogated, certain surface markers like VSIG4 and transcription factors necessary for KC function are down-regulated, resulting in altered cell morphology. Without ALK1, the cells exhibit a more inflammatory phenotype, and monocyte-derived macrophages eventually replace KCs in the liver. Similarly, the KO of BMP-9 and -10 in HSCs in mice led to reduced numbers of resident macrophages in the liver, suggesting that embryonic KCs were lost and replaced by monocyte-derived macrophages [40]. These changes consequently hindered the KCs’ capacity to collect pathogens, including Listeria monocytogenes, underscoring the significance of BMP-9/ALK1 signaling in the livers’ macrophage-mediated defense mechanisms [41]

In a mouse model of nonalcoholic steatohepatitis (NASH) caused by methionine- and choline-deficient (MCD) diet feeding, adenoviral overexpression of BMP-9 promoted inflammatory responses and supported the M1-polarization of macrophages [29,42]. In line with this, co-injections of bacterial toxin (LPS) and recombinant BMP-9 in mice *in vivo*, as well as such co-stimulation of human macrophages *in vitro*, at least partially led to synergistic enhancement of pro-inflammatory responses [14]. These results indicate that by directly acting on macrophages, BMP-9 might exert pro-inflammatory effects that are possibly needed for proper host defense mechanisms in the liver. If BMP-9 acts in a similar fashion on all macrophages of the body (not only on KCs) remains to be investigated further.

### Non-ALK1 signaling

While ALK1 is the most studied high-affinity receptor for BMP-9, signaling can also occur through different receptor complexes. BMP-9 can activate non-ALK1-dependent pathways, e.g., via binding to ALK2, at higher concentrations, potentially leading to distinct signaling outcomes and functional consequences. If ALK1 is absent, BMP-9 can bind to ALK2, again triggering Smad 1/5/8 signaling, e.g., in cancerous and myoblast cells [32,43], but also in primary hepatocytes [4]. Since both ALK1 and ALK2 are activating mainly the same downstream signal (Smad-1/5/8 phosphorylation leading to, e.g., ID-protein induction), it can be assumed that both receptors act in a fully redundant fashion. The existence of different receptors probably represents a mechanism to fine-tune BMP-9 responses: if ALK1 is present, ALK2 (or other BMP-ALKs) becomes irrelevant because all BMP-9 will be captured by ALK1. On the other hand, absence of ALK1 still does not exclude any responsiveness; it is only a stoichiometric question—when BMP-9 levels reach a sufficiently high concentration, non-ALK1 pathways will still be activated (see also Figure 1). On the other hand, ALK2 can also bind other ligands besides BMP-9 and the ligand-receptor complexes that form on the cell surface can additionally include co-factors or co-receptors like endoglin or BMPER (see below) which in turn harbor differential affinities to the ligands and/or receptors resulting in active or inactive complexes. This means that even though both ALK1 and ALK2 can principally activate the same downstream (Smad-) cascade, whether signaling is transduced or not depends on more factors and is probably in addition regulated in a cell type-specific fashion.

### Non-Smad pathways

BMP-9 also triggers noncanonical pathways, such as the PI3K/Akt and MAPK pathways, which affects a number of cellular functions, including survival and proliferation. For example, BMP-9 directly affects human osteoclasts by enhancing bone resorption and protecting them against apoptosis. Knocking down the BMPR-II receptor abrogated BMP-9-induced ERK signaling, as well as the increase in bone resorption in these cells, although with 150 ng/ml the amount of BMP-9 used in this study was rather unphysiologically high [43]. In Hep3B cells (a human hepatocellular carcinoma (HCC) cell line), BMP-9 contributes to growth-promoting effects by activating the p38 MAPK pathway [44]. These non-Smad pathways are typically activated in parallel to the Smad cascade, but it remains rather less well understood how BMP-9 mechanistically mediates, e.g., phosphorylation of ERK or p38.

### The co-receptor endoglin

Endoglin, also known as CD105, is a membrane glycoprotein which is primarily found on ECs. It acts as a co-receptor for ligands of the TGF-β family and plays a significant role in angiogenesis and vascular remodeling. The mode of action of soluble endoglin most likely involves the capturing of BMP-9 from the blood, promoting its binding to ALK1 on a cell surface followed by release of endoglin from the complex and its replacement with the type II receptor which results in an active signaling complex [45]. Thereby, endoglin can generally be considered as a promoter of BMP-9 signaling:

Endoglin acts as a co-receptor for BMP-9, and their interaction has been extensively studied, especially in the context of vascular biology and endothelial function [46,47]. This interaction is essential for promoting vascular quiescence and protecting ECs from apoptosis [36,37,48]. BMP-9 signaling through ALK1 is enhanced by endoglin, leading to the activation of the Smad1/5/8 pathway, resulting in inhibited EC migration and growth and supporting expression of endothelial differentiation markers [49] which in turn regulates angiogenesis and endothelial homeostasis [37,49]. Loss of BMP-9 or mutations in the endoglin gene (ENG) can lead to vascular disorders such as arteriovenous malformations (AVMs) or hereditary hemorrhagic telangiectasia (HHT) [36,46,49,50]. In cancer cells, but not ECs, soluble endoglin (sENG) can modulate BMP-9 signaling leading to sENG-mediated inhibition of BMP-9-induced apoptosis [36,46,48]. Cancer cells seem to sometimes use endoglin and ALK1 to trap BMP-9 protein: sENG binds to BMP-9 with high affinity and thereby blocks the type II receptor binding site on BMP-9, resulting in ALK1-mediated binding of BMP-9 to the cell surface but without any signal transduction being initiated. This was, for example, described in blood cancer cells, but for unknown reasons, this does not happen in vascular ECs; here, sENG does not inhibit signaling [36]. Similarly, in squamous cell carcinoma cell lines, it was reported that overexpression of endoglin reduced BMP-9 mediated Smad signaling [51]. This might represent a mechanism of cancer cells to neutralize tumor-suppressive BMP-9 signaling and thereby support its own growth. Nevertheless, an antagonistic action of endoglin was also reported for normal human cardiac fibroblasts, where endoglin reduced basal as well as TGF-β induced expression of BMP-9 [10].

In this context, it needs to be pointed out that although endoglin was initially identified on ECs, more recent work has shown that a wide variety of other cell types, including tumor cells, immune cells, and fibroblasts can as well express endoglin [52].

## Mechanisms of BMP-9-induced liver fibrogenesis

**Figure 2:**
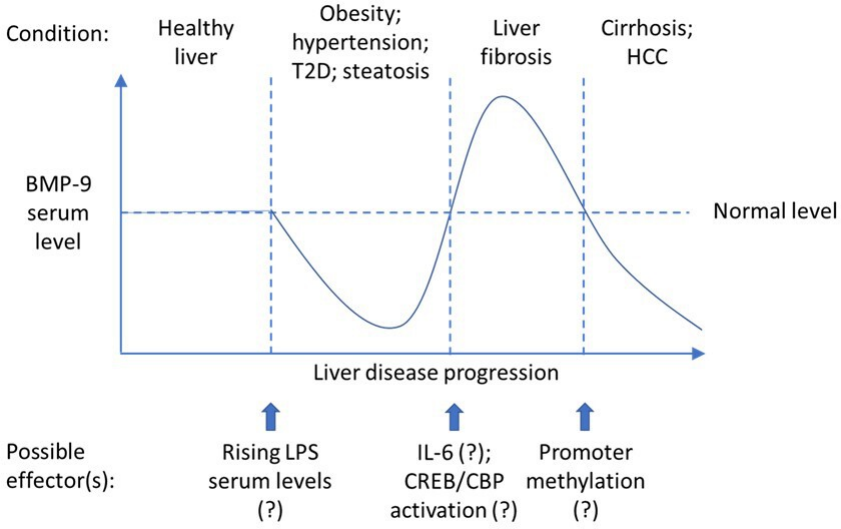
Schematic projection of the changes of BMP-9 serum levels in correlation to the progression of hepatic disease. See text for details. It remains to be proven in humans whether the mentioned effectors proposed to cause elevation or decrease of BMP-9 serum levels are indeed relevant *in vivo*. CREB: cAMP response element binding protein, CBP: CREB-binding protein, T2D: diabetes mellitus type 2, HCC: hepatocellular carcinoma

It is well accepted nowadays that BMP-9 represents an important cytokine for maintenance of normal liver function (summarized in [53]). Serum levels of BMP-9 seem to decrease initially with early stages of liver disease, like steatosis, but come up again with fibrogenesis. If disease progresses to cirrhosis, however, BMP-9 levels drop again (Table 1 and Figures 1 and 2). Interestingly, BMP-9 neutralization or knock-out resulted in reduced liver fibrosis in experimental mouse models [4,16,29] supporting pro-fibrotic actions of BMP-9 in chronic liver damage *in vivo*. Indeed, BMP-9 stimulated HSC proliferation and migration *in vitro*, but without directly enhancing expression of activation markers [4]. Others have reported a direct HSC-activating effect of BMP-9 using the human cell line LX-2 in *in vitro* experiments [29]. However, this direct activation was determined mainly by measuring enhanced expression of aSMA, not other typical markers like collagen, and with up to 50 ng/ml rather high amounts of recombinant BMP-9 were used. Nevertheless, the pro-proliferative and pro-migratory effects of BMP-9 and its pro-fibrotic actions *in vivo* were clearly reproduced in this work. In summary, BMP-9 is expressed in both quiescent HSCs and myofibroblasts, but in contrast with TGF-β, it does not directly activate the cells. The underlying mechanism of the pro-fibrotic effect of BMP-9 could involve its anti-regenerative functions. It was described that BMP-9 expression *in vivo* gets transiently down-regulated under conditions where regenerative actions, e.g., proliferation and migration of hepatocytes, are needed. By supporting cellular differentiation, high levels of BMP-9 would antagonize de-differentiation, which in turn is transiently needed for efficient hepatic regeneration and wound healing. Also, by stabilizing the differentiated phenotype of LSECs, BMP-9 antagonizes capillarization. The latter process involves de-differentiation of this highly specialized type of ECs. This also explains why lack of BMP-9 seems to result in a low degree of fibrosis in 129/Ola-mice [54], which is probably the result of de-differentiation of LSECs in the absence of the homeostatic effect of BMP-9 (see also Figure 1). This process of capillarization represents a well-described promoter of pro-fibrogenic mechanisms in the liver, including HSC activation [55].

In line with this concept of BMP-9 hindering regenerative processes in the liver, in a mouse model of cholestasis (induced by DDC), lack of BMP-9 resulted in enhanced liver regeneration [56].

## Actions of BMP-9 in metabolic syndrome/obesity/diabetes

BMP-9 obviously protects from obesity and metabolic derailment: BMP-9 directly reduces serum glucose levels [27]. Furthermore, BMP-9 promoted the ability of insulin to activate the insulin receptor/phosphoinositide 3-kinase PI3K/Akt pathway in the hypothalamus, and this ameliorated hepatic glucose metabolism and IR in mice [12]. The authors of this work also showed that adenoviral overexpression of hypothalamic BMP-9 led to reduced weight gain in the high-fat diet (HFD) feeding mouse model, implying that systemic BMP-9 acts protective against obesity and its consequences in mice. In line with such protective BMP-9 effects, serum levels of BMP-9 were significantly decreased in humans developing obesity-related complications like IR, type 2 diabetes, or fatty liver disease (Table 1).

## Effects of BMP-9 on the heart

As mentioned above, circulating BMP-9 protein levels were described to increase in patients with heart failure [10] or myocardial infarction [31], implying a functional role for BMP-9 in diseases of the heart. Indeed, in a mouse model of heart failure, promoting BMP-9 activity attenuated cardiac fibrosis and increased levels of BMP-9 limited TGF-β1 signaling and type I collagen synthesis in the left ventricle *in vivo* [10]. In line with this, recent findings using a mouse model of myocardial infarction again demonstrated a protective function of BMP-9 *in vivo* [31].

## BMP-9s’ roles in cancer

Being a homeostasis factor, BMP-9 is often described to promote and stabilize cellular differentiation, which most likely affects the transition of differentiated nonmalignant cells into dedifferentiated cancer cells.

However, the specific effects of BMP-9, e.g., on the formation of cancer stem cells, are diverse and depend on the different types of cancer cells.

Hepatocellular Carcinoma (HCC): To our knowledge, there is no notion so far of changed serum levels of BMP-9 in HCC patients compared with fully healthy individuals. However, it seems that HCC patients can be divided into subgroups of high versus low expressors based on protein [57,58] or serum levels [59]. Correlation analyses indicate that the subgroup having high BMP-9 expression in tumor tissues or serum correlates with poorer outcome [59] and expression levels of BMP-9 mRNA and protein were significantly associated with the T stages of the investigated tumors of a cohort of HCC patients [57]. Treatment with the BMP-receptor inhibitor LDN-212854, which inhibits both ALK1 and ALK2, suppressed HCC tumor growth by repressing ID1 and EpCAM *in vivo* in a xenograft mouse model of HCC [59]. BMP-9 promotes cancer stem cell properties in EpCAM-positive HCC cells through up-regulating the expression of ID1 and activating the Wnt/β-catenin pathway. In conclusion, it might be speculated that at least certain sub-groups of HCC patients would potentially profit from BMP-9 targeted therapies.

Colorectal Cancer (CRC): The expression of BMP-9 is elevated during the transition from normal mucosa to adenocarcinoma, which potentially promotes the formation of cancer stem cell traits via non-Smad signaling pathways [60]. In contrast, our previous findings point to a tumor-suppressive function of BMP-9 in CRC by inducing ID1 whose expression is positively correlated with better survival in patients [61]. We further showed that BMP-9 potentially stabilizes the gut barrier and thereby protects from the toxic actions of LPS. On the other hand, LPS itself can possibly down-regulate BMP-9 also in the gut, meaning that transitioned LPS, after exceeding a certain threshold, might lead to reduced local BMP-9 which would further weaken the gut barrier and promote disease progression.

Breast and prostate cancer: In HER2-positive breast cancer and in prostate cancer, BMP-9 again exerts tumor suppressive effects by inhibiting apoptosis, suggesting a possible reduction in the formation of cancer stem cell activity [62]. One study further found that the expression of BMP-9 is significantly lower in breast cancer tissue compared with the adjacent nontumor tissues. The study involved 23 clinical breast cancer tumor tissues and the positive rate of BMP-9 was reported to be only 17.4% in tumorous parts but 65.2% in paracancerous tissue [63]. In particular, in breast cancer cells, BMP-9 is reported to inhibit the proliferation through several mechanisms, which include apoptosis induction in MDA-MB-231 and reduced proliferation upon overexpression of BMP-9 [64]. BMP-9 can also lead to down-regulation of leptin expression and reduce phosphorylation of STAT3, ERK1/2, and AKT, all being mechanisms that are involved in cell growth and survival pathways in breast cancer [65]. PI3K/Akt pathway is down-regulated by BMP-9 and this is crucial for the proliferation and survival of breast cancer cells [63]. In line with these findings, in a syngeneic immunocompetent orthotopic mouse model of spontaneous metastatic breast cancer (E0771), it was impressively demonstrated that loss of BMP-9 (but not BMP-10) resulted in increased tumor growth and formation of lung metastases [66]. In contrast, neutralization of BMP-9 (and -10) using ALK1Fc reduced prostate cancer cell proliferation and tumor growth *in vivo* in an orthotopic transplantation model, as well as in the human patient-derived xenograft BM18 [67].

These mechanisms collectively contribute to a complex role of BMP-9 as a potential tumor suppressor in some types of cancer whereas promoting the growth of others.

## BMP-9 inhibitors

### Inhibitory Smads, Smad6 and 7

BMP-9 signaling is antagonized by the inhibitory Smads (I-Smads), Smad6 and Smad7. While Smad7 inhibits both TGF-β and BMP-induced signaling, including BMP-9, Smad6 preferentially inhibits BMP signaling by interacting with BMP type I receptors such as ALK-1,–3, and -6 [68,69]. The subcellular localization of I-Smads differs in their local distribution among cytoplasm and nucleus. Smad7 can be found in both cytoplasm as well as nucleus, mostly simultaneously and particularly at the invasive front of carcinomas [70]. The dual localization of Smad7 allows it to effectively inhibit signaling pathways by interacting with the receptors in the cytoplasm and potentially also influencing its transcriptional activity in the nucleus. Similarly, Smad6 is also localized in both the nucleus and cytoplasm, as was shown for P19- and HeLa cells, and in the nucleus, it functions as a transcriptional repressor of BMPs by recruiting co-repressors such as CtBP [71]. Thereby, Smad6, being itself an immediate-early target gene of BMP signaling, can principally act on three levels: it inhibits R-Smad activation at the membrane, counteracts R-Smad/Smad4 heteromerization in the cytoplasm, and can also repress transcription of target genes in the nucleus.

Both Smad6 and Smad7 can suppress BMP-9 signaling by restricting phosphorylation and nuclear translocation of R-Smads [68,72]. In comparison with Smad6, the N-domain of Smad7 is more effective at facilitating interaction with type I receptors. Smad7’s Mad Homology 2 (MH2) domain interacts with this N-domain, which is crucial for its inhibitory action. The Smad7 N-domains’ capacity to strengthen its binding to the type I receptor is what causes this improved interaction compared with Smad6 [73,74]. The Smad6’s N-domain performs this activity less efficiently. This distinction might be one explanation why Smad7 is usually more effective than Smad6 at blocking signaling pathways, including those mediated by TGF-β and BMPs [73]. One study reported that the MH2 domain (amino acids 332–496) of Smad6 binds directly to Smad4 [75] and thereby the Smad1/5/8-Smad4 complex that is required to transmit BMP-9 signals to the nucleus cannot form [68,76].

To further inhibit BMP-9 signaling, Smad6 and Smad7 can also recruit ubiquitin ligases such as Smurf1 and Smurf E3, respectively, which lead to degradation of type I BMP receptors [68]. Additionally, Smurfs can directly interact with R-Smads and lead to their degradation [77,78]. In ECs treated with BMP-9, Smad6 inhibition may increase the activity of the non-Smad pathways, like p38 MAPK pathways. Increased p38 MAPK phosphorylation is involved in BMP-9 induced processes such as endothelin-1 (ET-1) release. ET-1 is a potent vasoconstrictor involved in vascular functions and is induced by BMP-9 in ECs [76,79].

### BMPER

Crossveinless 2 (CV2), also known as BMP endothelial precursor cell-derived regulator (BMPER), is a secreted glycoprotein that binds directly to BMPs and modulates their function in a dose-dependent manner. BMPER directly binds to BMP-9 and thereby interferes with its receptor binding site, resulting in strong inhibition of BMP-9/ALK1 signaling [80]. This interaction between BMPER and BMP-9 prevents receptor activation, thereby affecting processes like the development of the vascular endothelium [80,81]. In summary, BMPER acts as an extracellular modulator of the BMP signaling pathway, but its relevance, especially with regard to BMP-9 in humans, remains to be supported further. Mao et al. [82] made the interesting observation that the delivery of BMPER recombinant protein or its overexpression alleviated insulin resistance and hyperglycemia in high-fat diet-fed mice and Lepr-(db/db) diabetic mice. As described above, systemic BMP-9 itself also acts protective against obesity and its consequences in mice. If BMPER inhibited the effects of BMP-9 *in vivo*, one would expect the opposite outcome after BMPER overexpression, i.e., increased obesity and enhanced susceptibility to diabetes. Further investigations are clearly needed here.

### Noggin

In Xenopus embryos that had been artificially ventralized, the glycoprotein noggin was able to restore a normal dorsal-ventral body axis [83]. Mammalian noggin is highly similar to Xenopus noggin and seems to play important roles in the nervous system [84]. Functionally, it was described that noggin can inhibit BMP signaling by blocking the molecular interfaces of the binding epitopes for both type I and type II receptors [85]. Thereby, the Smad pathway triggered by BMPs is often inhibited by noggin. Nevertheless, BMP-9 induces ID1 expression even in the presence of noggin, and this is due to its resistance to noggin’s inhibitory effects. This unique property of BMP-9 compared with other BMPs has, e.g., been observed in studies involving CRC organoids in our lab, where BMP-9 stimulated ID1 expression despite high levels of noggin [61] but also earlier by others in osteoblasts [86]. This resistance to noggin makes BMP-9 a potential therapeutic target, especially in the field of bone regeneration (summarized in [87]), but potentially also in cancer settings like CRC.

## Cross-talk between BMP-9 and TGF-β

Of particular importance is BMP-9’s intricate relationship with the TGF-β pathway, as both are members of the same superfamily of cytokines and both play crucial roles in processes like cancer development and progression. The cross-talk between BMP-9 and TGF-β involves complex interactions that influence various cellular processes. Despite sharing Smad4 as a signaling component, there is limited mutual inhibition between TGF-β and BMP pathways, with more evidence of activating cross-talk from TGF-β to BMPs in general rather than vice versa [88]. The interaction is highly context-dependent, influenced by cell type and developmental stage, and involves multiple signaling pathways [89]. For instance, in MSCs, BMP-9 enhances osteogenic differentiation by inducing TGF-β1 expression, which has a biphasic effect on this process. This involves the activation of Smad1/5/8 and interaction with Smad4, crucial for osteogenesis [34,90]. In ECs, BMP-9 does not significantly induce TGF-β1 expression, but it regulates other pathways like the SDF1/CXCL12 chemokine axis, thereby influencing angiogenesis and vascular remodeling. However, the relation between the two cytokines is complicated as evidenced by the finding that *in vitro* stimulation of ECs with either BMP-9 or TGF-β alone led to inhibition of tube formation and proliferation, but when BMP-9 and TGF-β were administered in combination, a stimulatory effect was observed [91].

BMP-9 signaling can involve ALK1 and endoglin, both affecting EC function [92,93] and a complex cross-talk exists between the BMP-9 and TGF-β receptors, including even the possible formation of ALK1/ALK5 heterodimers whose activation by TGF-β can be enhanced by endoglin while an ALK5 homodimer would be inhibited by endoglin [94].

BMP-9 mediated differentiation of MSCs into osteoblasts was significantly enhanced when the cells were co-stimulated with TGF-β [95]. Interestingly, in MSCs, adenoviral overexpression of BMP-9 also directly induced TGF-β expression itself [96]. The combination of BMP-9 and TGF-β also resulted in increased alkaline phosphatase activity and an elevated expression of osteogenic markers like COL1 and OPN, and this appeared to be more significant than when using BMP-9 or TGF-β alone [95,96].

In summary, the two cytokines can principally enhance or inhibit each other’s activity, or even revert the responses when acting on a given cell in combination. The underlying factors and mechanisms are only partially understood and definitely need to get better characterized in the future.

## Clinical trials targeting the BMP-9 pathway(s)

Pulmonary arterial hypertension (PAH) is a complex disease of the lungs that includes vascular abnormalities, and it has been found that in 70-80% of the heritable PAH cases, the BMPRII (which is also bound by BMP-9) is mutated [97], implying that the BMP-pathway is relevant for the pathogenesis of this disease. Another disease that involves vascular malformations in the lungs (and other organs) is hereditary hemorrhagic telangiectasia (HHT), which is mainly caused by mutations in ALK1 [98] and endoglin [99]. Together, these observations imply that the BMP-9 pathway might represent a good therapeutic target in lung diseases, and indeed, clinical trials which target the BMP-9 pathway focused especially on pulmonary diseases. BMP-9 has demonstrated promising results in reversing pulmonary arterial hypertension (PAH) [100] and acute respiratory distress syndrome (ARDS) [101]. The company, Morphogen-IX, has developed a mutated version of the BMP-9 protein to generate a series of amino acid substitutions in the receptor binding domains of BMP-9, aiming to remove osteogenic activity but to retain endothelial protective signaling. The resulting protein called MGX292 is currently tested as a drug to enhance vascular stability and prevent endothelial cell apoptosis. However, no data for effective use of this protein in the clinic are available yet.

Recent developments aiming at finding inhibitors targeting the BMP-9/ALK1 axis include the investigation of Dalantercept (ACE-041) and PF-03446962. Dalantercept is an ALK1-Fc fusion protein that acts as a ligand trap and leads to capture of BMP-9 and BMP-10. Dalantercept is tested already in clinical trials targeting advanced cancers, and it shows some promise in combination therapies with agents like sorafenib and regorafenib [94]. However, in a study with rather small numbers of participants where Dalantercept was tested for the treatment of advanced renal cell carcinoma (NCT01727336), it did not appear to improve treatment-related outcomes [102]. Additional studies were performed on patients with other types of cancers (HCC [103], ovarian cancer [104], endometrial cancer [105], head and neck cancer [106]), but also here the results were not promising.

PF-03446962, a fully human antibody against ALK1 that inhibits BMP-9 induced signaling, has demonstrated efficacy in preclinical studies. This antibody inhibited endothelial cell sprouting but without directly interfering with VEGF-mediated effects [107]. Hu-Lowe et al. postulated that ALK1 signaling, similar to VEGF, acts pro-angiogenic, and they describe that its inhibition resulted in decreased vessel density and improved antitumor efficacy when combined with bevacizumab (an anti-VEGF antibody) [108]. A direct action of BMP-9 was not (yet) considered in that study, and a cross-talk between TGFβ/ALK1 and VEGF/bFGF pathways was suggested. Nowadays, it is well accepted that BMP-9 binds to ALK1 with much higher affinity than TGF-β, but the individual constellation of ligands and receptors within the microenvironment of a growing tumor probably dictates a pro- or anti-angiogenic outcome of blocking ALK1.

PF-03446962 is also undergoing clinical evaluation in cancer settings, but so far, it demonstrated rather insufficient efficacy or high toxicity in phase I and II studies [94]. As suggested for HCC, maybe only certain subtypes of cancers should be treated with anti-ALK1 strategies, and the criteria to define these subtypes still need to be investigated in more detail.

## Summary and future perspectives

This review consolidates current understanding of BMP-9, a pleiotropic cytokine with functions far beyond bone formation. BMP-9 is primarily synthesized by HSCs in the liver and regulates angiogenesis, macrophage polarization, and hepatocyte functions via interactions with diverse liver cell types. While BMP-9 predominantly signals through the ALK1 receptor—critical for endothelial quiescence and Kupffer cell identity—alternative signaling via ALK2 and non-Smad pathways (PI3K/Akt, MAPK) exists, sometimes additionally modulated by the co-receptor endoglin. BMP-9 serum levels, influenced by factors like cold exposure, IL-6, or LPS, are altered in conditions such as adiposity, diabetes, fatty liver disease, but also fibrosis and multiple myeloma. It remains to be investigated how the BMP-9 pathway(s) can best be targeted for therapy or to stabilize tissue homeostasis effectively in humans. However, complete blockage of BMP-9 signaling will most likely not be the best strategy. It might rather be tested if optimization of BMP-9 levels in a personalized manner represents perhaps a more promising approach. For this, we would first need some kind of rapid test to analyze a patients’ individual BMP-9 serum levels and then—if it is outside the optimal range—pharmaceutically adjust it. Today, we are still far away from such application, and we need a lot more research on this interesting and multifaceted cytokine.

## Abbreviations

ALK: activin-like kinase
ARDS: acute respiratory distress syndrome
BMP: bone morphogenetic protein
BMPER: BMP endothelial precursor cell-derived regulator
BP: blood pressure
CRC: colorectal cancer
CV2: crossveinless 2
EC: endothelial cell
EMT: epithelial-mesenchymal transition
ENG: endoglin
EPC: endothelial progenitor cell
ET-1: endothelin-1
EndoMT: endothelial-mesenchymal transition
HCC: hepatocellular carcinoma
HFD: high-fat diet
HHT2: hereditary hemorrhagic telangiectasia type 2
HSC: hepatic stellate cell
IR: insulin resistance
KC: kupffer cell
KO: knock-out
LDL: low-density lipoprotein
LPS: lipopolysaccharide
LSEC: liver sinusoidal endothelial cell
MAPK: mitogen activated protein kinase
MCD diet: methionine and choline-deficient diet
MH2: mad homology 2
MSC: mesenchymal stem cell
NAFLD: nonalcoholic fatty liver disease
NASH: nonalcoholic steatohepatitis
PAH: pulmonary arterial hypertension
R-Smads: receptor-regulated Smads
SBE: smad binding elements
TGF: transforming growth factor
VEGF: vascular endothelial growth factor
bFGF: basic fibroblast growth factor
sENG: soluble endoglin
